# Supplementary material for: In Vitro Synergism of Penicillin and Ceftriaxone against Enterococcus faecalis
Source: Microorganisms. 2021 Oct 14;9(10):2150. doi: 10.3390/microorganisms9102150 (PMC8541343; doi:10.3390/microorganisms9102150)
Supplement: Supplementary file 1 [file microorganisms-09-02150-s001.zip › microorganisms-1371730-supplementary.pdf]

Supplementary material microorganisms-1371730

Table S1: Antibiotic susceptibility results determined by Vitek 2 (bioMerieux, France). Susceptibility breakpoints are based on EUCAST breakpoints. S = susceptible, R = resistant, I = intermediate. N.A. = not available.

Isolate	vancomycin	teicoplanin	linezolid	quinupristin/ dalfopristin	moxifloxacin	levofloxacin	trimethoprim/ sulfamethoxazol	gentamicin/high level	tigecyclin
5187	S	S	S	R	S	S	R	R	S
245	S	S	S	R	S	R	N.A.	S	S
ATCC	N.A.	N.A.	N.A.	N.A.	N.A.	N.A.	N.A.	N.A.	N.A.
6747	S	S	S	R	S	S	R	S	S
8669	S	S	S	R	S	S	R	S	S
9367	S	S	S	N.A.	S	I	R	S	S
1653	S	S	S	N.A.	N.A.	S	S	N.A.	S
848	S	S	S	R	S	I	R	S	S
5597	S	S	S	R	S	S	R	S	S
6886	S	S	S	R	S	S	R	S	S
281	S	S	S	R	S	S	R	S	S
67230	S	S	S	R	S	I	R	S	S
2164	S	S	S	R	S	I	R	S	S
4497	S	S	S	N.A.	S	I	R	R	S
905	S	S	S	R	S	S	R	S	S
6037	S	S	S	N.A.	R	R	R	N.A.	S
7183	S	S	S	R	R	R	R	R	S
3043	S	S	S	R	R	R	R	R	S
8653	S	S	S	R	R	R	R	R	S
3062	S	S	S	R	R	R	R	R	S
9190	S	S	S	R	R	R	R	R	S
404	S	S	S	N.A.	N.A.	S	N.A.	S	S
11223	S	S	S	N.A.	S	I	R	S	S
22424	S	S	S	R	N.A.	S	N.A.	S	S
26786	S	S	S	R	N.A.	S	N.A.	S	S
10485	S	S	S	N.A.	N.A.	N.A.	I	S	S

10021	S	S	S	N.A.	R	R	R	R	S
13703	S	S	S	N.A.	S	I	R	S	S
7914	S	S	S	R	R	R	N.A.	R	S

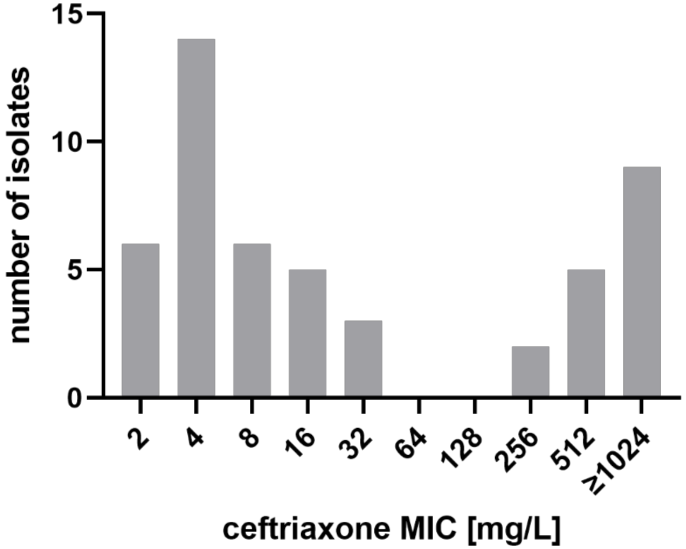

Figure S1: Ceftriaxone MIC distribution data for the *E. faecalis* strain collection at the Institute of Infectious Diseases and Infection Control, Jena University Hospital, Germany.
